# Nationwide outcomes of 1000 robotic pancreatoduodenectomies across the four phases of the learning curve

**DOI:** 10.1093/bjs/znaf210

**Published:** 2025-11-06

**Authors:** Anouk M L H Emmen, Bram L J van den Broek, Tessa E Hendriks, Olivier R Busch, Bert A Bonsing, Marie L Cappelle, Peter-Paul L O Coene, Sebastiaan Festen, Erwin van der Harst, Ignace H J T de Hingh, Cees J H M van Laarhoven, Daan J Lips, Joost Sprakel, Misha D P Luyer, J Sven D Mieog, Hjalmar C van Santvoort, George van der Schelling, Jan H Wijsman, Gijs A Patijn, Roeland F de Wilde, Maurice J W Zwart, Wouter J M Derksen, I Quintus Molenaar, Bas Groot Koerkamp, Marc G Besselink, Members of the Dutch Institute for Clinical Auditing and the Dutch Pancreatic Cancer Group, Freek Daams, Geert Kazemier, Khe Tran, Roel Haen, Alexander Vahrmeijer, Volkert Huurman, Robert Jan Schipper, Ronald van Dam, Jennifer Schreinemakers, Lieke Brouwer-Hol, Brigitte C M Haberkorn, Mike Liem, Wouter te Riele, Jeroen Hagendoorn, Martijn Stommel, Tom M Karsten

**Affiliations:** Department of Surgery, Amsterdam UMC, location University of Amsterdam, Amsterdam, The Netherlands; Cancer Centre Amsterdam, Amsterdam, The Netherlands; Department of Surgery, Erasmus MC Cancer Institute, Erasmus University Medical Centre, Rotterdam, The Netherlands; Department of Surgery, Amsterdam UMC, location University of Amsterdam, Amsterdam, The Netherlands; Cancer Centre Amsterdam, Amsterdam, The Netherlands; Department of Surgery, Leiden University Medical Centre, Leiden, The Netherlands; Dutch Institute for Clinical Auditing, Leiden, The Netherlands; Department of Surgery, Amsterdam UMC, location University of Amsterdam, Amsterdam, The Netherlands; Cancer Centre Amsterdam, Amsterdam, The Netherlands; Department of Surgery, Leiden University Medical Centre, Leiden, The Netherlands; Department of Surgery, Erasmus MC Cancer Institute, Erasmus University Medical Centre, Rotterdam, The Netherlands; Department of Surgery, Maasstad Hospital, Rotterdam, The Netherlands; Department of Surgery, OLVG, Amsterdam, The Netherlands; Department of Surgery, Maasstad Hospital, Rotterdam, The Netherlands; Department of Surgery, Catharina Hospital, Eindhoven, The Netherlands; Department of Surgery, Radboud UMC, Nijmegen, The Netherlands; Department of Surgery, Medisch Spectrum Twente, Enschede, The Netherlands; Department of Surgery, Medisch Spectrum Twente, Enschede, The Netherlands; Department of Surgery, Catharina Hospital, Eindhoven, The Netherlands; Department of Surgery, Leiden University Medical Centre, Leiden, The Netherlands; Department of Surgery, Regional Academic Cancer Centre Utrecht, UMC Utrecht Cancer Centre & St Antonius, Utrecht, The Netherlands; Department of Surgery, Amphia Hospital, Breda, The Netherlands; Department of Surgery, Amphia Hospital, Breda, The Netherlands; Department of Surgery, Isala, Zwolle, The Netherlands; Department of Surgery, Erasmus MC Cancer Institute, Erasmus University Medical Centre, Rotterdam, The Netherlands; Department of Surgery, Amsterdam UMC, location University of Amsterdam, Amsterdam, The Netherlands; Cancer Centre Amsterdam, Amsterdam, The Netherlands; Department of Surgery, Regional Academic Cancer Centre Utrecht, UMC Utrecht Cancer Centre & St Antonius, Utrecht, The Netherlands; Department of Surgery, Regional Academic Cancer Centre Utrecht, UMC Utrecht Cancer Centre & St Antonius, Utrecht, The Netherlands; Department of Surgery, Erasmus MC Cancer Institute, Erasmus University Medical Centre, Rotterdam, The Netherlands; Department of Surgery, Amsterdam UMC, location University of Amsterdam, Amsterdam, The Netherlands; Cancer Centre Amsterdam, Amsterdam, The Netherlands

## Abstract

**Background:**

Robotic pancreatoduodenectomy (RPD) is increasingly being implemented to enhance patient recovery, but it is unclear what happens to patient outcomes after the initial learning curve. The aim of this study was to evaluate the first 1000 consecutive RPD performed in the Netherlands.

**Methods:**

A nationwide analysis of patients who underwent RPD in 13 centres (March 2016–August 2023) from the Dutch Pancreatic Cancer Audit was performed. Patients were grouped based on published learning curve cut-offs (phases 1–4): 1–15, 16–62, 63–84, and >84 RPD per centre respectively. Outcomes were compared between the four learning curve phases. Ideal Outcome rates were used to compare outcomes between centres.

**Results:**

Overall, 1000 patients after RPD were included. The conversion rate was 10.1%, the rate of Clavien–Dindo complications of grade ≥III was 41.3%, the rate of postoperative pancreatic fistula of grade B/C was 24.4%, and the rate of in-hospital/30-day mortality was 3.9%. Of the patients, 71.1% had a high updated alternative fistula risk score. Improvements between the phases were found for five outcomes: median operating time (420, 360, 349, and 369 min respectively; *P* < 0.001), conversion rate (21.7%, 10.0%, 2.8%, and 7.5% respectively; *P* < 0.001), rate of delayed gastric emptying (DGE) of grade B/C (32.3%, 22.6%, 15.4%, and 20.2% respectively; *P* = 0.003), reoperation rate (9.9%, 11.3%, 9.8%, and 4.9% respectively; *P* = 0.026), and median duration of hospital stay (12, 11, 10, and 10 days respectively; *P* = 0.035). The rate of Clavien–Dindo complications of grade ≥III and the rate of in-hospital/30-day mortality remained stable. The Ideal Outcome rate (mean 47%) did not differ between centres.

**Conclusion:**

Across four learning curve phases in a nationwide cohort, improvements were observed for operating time, conversion rate, rate of DGE of grade B/C, reoperation rate, and duration of hospital stay.

## Teaser

A nationwide analysis of 1000 patients who underwent robotic pancreatoduodenectomy (RPD) in 13 centres (March 2016–August 2023) was performed. Outcomes were compared between patients in the four learning curve phases (1–15, 16–62, 63–84, and >84 RPD), determined per centre. Improvements were seen for median operative time, conversion rate, delayed gastric emptying grade B/C, reoperation rate, and median hospital stay. Between the four phases, the rate of Clavien–Dindo complications grade ≥III and the rate of in-hospital/30-day mortality remained stable. The nationwide rate of Ideal Outcome rate remained stable.

## Introduction

Robotic pancreatoduodenectomy (RPD) is increasingly being implemented in high-volume centres worldwide with the aim of enhancing patient recovery compared with the open approach. Conversely, a recent phase 2b randomized trial^1^ and a recent phase 2/3 randomized trial^2^ only reported limited benefits for patients who undergo RPD compared with open pancreatoduodenectomy (OPD). These outcomes in expert centres fuel concerns regarding the outcomes and benefits of RPD when implemented on a nationwide scale.

It is well known that obtaining sufficient proficiency in RPD is associated with a considerable learning curve effect^3–6^. Whereas outcomes for OPD have largely stabilized after decades of developments, it is unclear what happens to the outcomes after RPD after the initial learning curve, especially on a nationwide scale. Moreover, it is unknown whether differences in outcomes exist between centres, especially for those that have completed the same training programme. This could have implications for future training programmes and might influence the interpretation of recently published randomized trials.

In the interval 2016–2019, the Dutch Pancreatic Cancer Group (DPCG) implemented RPD through a nationwide training programme overseen by the University of Pittsburgh Medical Centre (UPMC)^7^. In this cohort, cut-offs for the RPD feasibility, proficiency, and mastery learning curves were determined at 15, 62, and 84 procedures respectively^5^. Now, almost 8 years after the start of this programme, >1000 RPD procedures have been performed in the Netherlands, providing a unique opportunity to assess the nationwide outcomes of consecutive RPD. In addition, it allows for a comparison between centres, most of which were trained in a highly standardized training. Therefore, the aim of this study was to report on the outcomes of 1000 consecutive RPD in the Netherlands, to identify nationwide changes in intraoperative and postoperative outcomes beyond the initial learning curve and among centres that have participated in training programmes.

## Methods

This nationwide multicentre retrospective cohort study included consecutive patients after RPD for all indications in the Netherlands (March 2016–August 2023). Data were retrieved from the nationwide Dutch Pancreatic Cancer Audit (DPCA), a mandatory audit in which all patients who undergo elective pancreatic resection are included. The minimum annual centre volume for pancreatoduodenectomy in the Netherlands is 20.

Patient baseline characteristics and intraoperative and postoperative outcomes during hospital stay and up to 30 days after surgery were collected by healthcare professionals in each participating centre. The study protocol was approved by the scientific committee of the DPCG^8^. As data were anonymized there was no need for informed consent or ethical approval according to Dutch regulations. Data were reported in compliance with the STROBE statement^9^.

This study was not pre-registered.

### System and centres

The da Vinci Xi Robotic Surgical System (Intuitive Surgical^®^, Inc., Sunnyvale, CA, USA) was used in all centres. Seven high-volume centres participated in the LAELAPS-3 RPD training programme^7^. The cut-off for high volume was set at 50 pancreatoduodenectomies per centre per year. Two additional centres participated in the more recent European LEARNBOT RPD training programme. Four centres received training independent of a study programme. In 11 of these 13 centres, RPD procedures were performed by two hepatopancreatobiliary (HPB) surgeons. In one centre, most procedures were performed by one HPB surgeon, with fellows (*n* = 5) who were trained as console surgeons, and, in one centre, a single-surgeon approach was used.

### Patient selection

No formal contraindications were applied for RPD. In the LAELAPS-3 training programme, it was advised to only perform RPD in the absence of portomesenteric vein and arterial involvement (assessed using a CT scan performed no more than 4 weeks prior), chronic and necrotizing pancreatitis, and BMI >35 kg/m^2^. With increased experience these contraindications became relative, especially once the ‘mastery’ cut-off point (84 RPD procedures) had been reached. One centre performed vascular reconstructions (segmental superior mesenteric vein, portal vein, and hepatic artery) in all phases of the learning curve.

### Definitions and outcomes

Baseline characteristics included age, sex, BMI (kg/m^2^), ASA grade, neoadjuvant chemo(radiation)therapy, and vascular and organ involvement. Surgical outcomes included operative time, intraoperative blood loss, conversion, pancreatic remnant aspect, pancreatic duct size (mm), postoperative complications (postoperative pancreatic fistula grade B/C^10^, post-pancreatectomy haemorrhage grade B/C^11^, delayed gastric emptying (DGE) grade B/C^12^, and bile leakage grade B/C^13^), severe postoperative complications (defined as Clavien–Dindo complications grade ≥III)^14^, 30-day readmission, 30-day reoperation, postoperative duration of hospital stay, R0 resection margin (that is ≥1 mm tumour-free margin), and in-hospital/30-day mortality.

### Nationwide improvements

Patients in each centre were ranked consecutively based on the RPD date. Trends over time were explored by chronologically ranking the RPD procedures per centre into the four phases of the learning curve (phases 1–4) based on previously published cut-offs: 1–15, 16–62, 63–84, and >84 RPD respectively^5^. Subsequently, data of all patients per phase were clustered and outcomes between the different phases were compared. A sensitivity analysis was performed in low-risk (pancreatic ductal adenocarcinoma (PDAC)) patients, excluding all non-PDAC patients.

### Centre-specific improvements

The outcomes with nationwide improvement seen over time were subsequently assessed for each individual centre to assess disparities. Outcomes could only be compared in the nine centres that had reached phase 2, as four centres only included patients in phase 1. Centre-specific improvement was defined as a significant improvement (*P* < 0.050) over the different phases.

### Outcomes in the most experienced centres

Centres that passed the ‘mastery’ cut-off of 84 RPD, and thus included patients in all four learning curve phases, were defined as the ‘most experienced centres’. These centres were compared for: the outcomes that showed nationwide improvement; operating time using a scatter plot with linear regression; and Ideal Outcome. Ideal Outcome has recently been defined as the absence of in-hospital mortality, Clavien–Dindo complications grade ≥III, postoperative pancreatic fistula grade B/C, reoperation, duration of hospital stay >75th percentile (in this study >20 days), and readmission after pancreatic resection^15^. In addition, a sensitivity analysis was performed to assess the presence of a plateau effect by excluding centres that performed <150 RPD.

### Statistical analysis

Data were analysed using SPSS^®^ (IBM, Armonk, NY, USA; version 26.0) and R’s programing environment (R Foundation for Statistical Computing, Vienna, Austria). Student’s *t* test, the Mann–Whitney *U* test, the chi-squared test, or Fisher’s exact test was used, as appropriate. Descriptive statistics were used for the analysis of the baseline characteristics, intraoperative outcomes, and postoperative outcomes for patients who underwent RPD. Results are reported as *n* (%) for binary or categorical variables and as mean(s.d.) or median (interquartile range (i.q.r.)) for continuous variables. *P* < 0.050 was considered statistically significant.

## Results

**Fig. 1:**
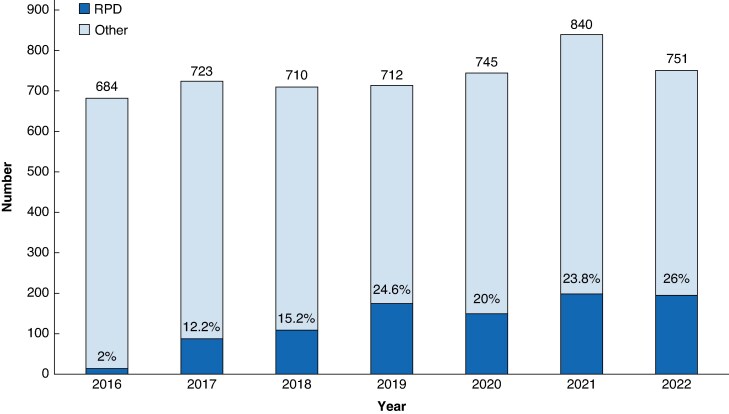
Nationwide annual volume of RPD in relation to other pancreatoduodenectomy RPD, robotic pancreatoduodenectomy.

**Fig. 2:**
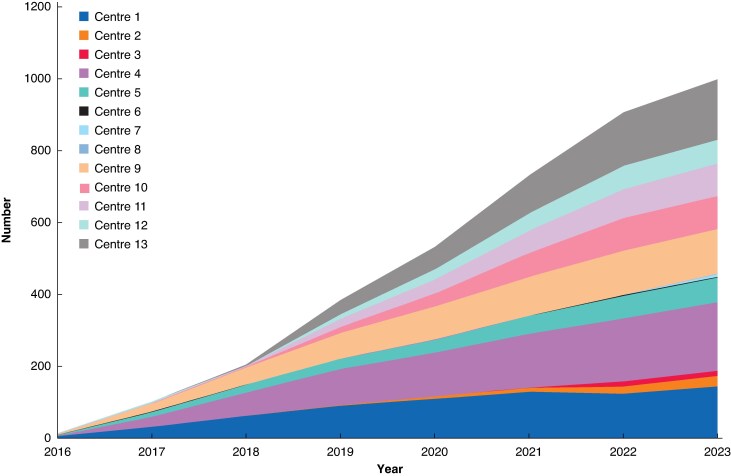
RPD per centre per year RPD, robotic pancreatoduodenectomy.

Overall, 1000 patients underwent RPD in 13 centres. The nationwide annual volume of RPD increased from 14 (2016) to 195 (2022); *P* < 0.001 (*Fig. 1*). The use of RPD among all pancreatoduodenectomies increased from 2% in 2016 to 26% in 2022. As the 13 centres started performing RPD at different points in time, their annual and overall volumes differed (*Fig. 2*). Therefore, these centres contributed to different phases of the learning curve: four centres performed <16 RPD (phase 1), one centre performed 16–62 RPD (phases 1 and 2), two centres performed 63–84 RPD (phases 1, 2, and 3), and six centres performed >84 RPD (phases 1, 2, 3, and 4).

The median patient age was 69 (i.q.r. 62–75) years and 562 patients (56.4%) were male. Most patients were ASA grade I–II (657 patients (66.8%)). The rate of pancreatic cancer was 29.7% and most patients (599 patients (71.1%)) had a high updated alternative fistula risk score (ua-FRS) (>20% risk of postoperative pancreatic fistula of grade B/C). The rate of vascular resection was 7.6% (76 patients); 70 of 76 patients underwent venous resections (wedge, 49 patients; segmental end-to-end without interposition graft, 20 patients; and segmental end-to-end with interposition graft, 1 patient). The rate of vascular resection remained relatively stable between 2017 (6.8%) and 2022 (9.2%); *P* = 0.500. The median operative time was 367 (i.q.r. 310–443) min and the conversion rate was 10.1% (101 patients). The rate of major morbidity (Clavien–Dindo complications grade ≥III) was 41.3% and the rate of in-hospital/30-day mortality was 3.9%. See *Table 1* and *Table 2*.

**Table 1:** Baseline and intraoperative characteristics

Variable	Overall RPD (*n* = 1000)	Phase 1, 1–15 RPD (*n* = 161)	Phase 2, 16–62 RPD (*n* = 389)	Phase 3, 63–84 RPD (*n* = 143)	Phase 4, >84 RPD (*n* = 307)	*P*
Age (years), median (i.q.r.)	69 (62–75)	68 (62–74)	70 (63–76)	70 (59–76)	69 (62–75)	0.550
Sex, male	562 (56.4)	97 (60.2)	217 (55.8)	76 (53.5)	172 (56.4)	0.681
BMI (kg/m^2^), median (i.q.r.)	25.1 (22.6–27.9)	25.3 (22.5–27.4)	25 (22.7–27.8)	25 (22.3–27.5)	25.2 (22.7–28.3)	0.816
ASA grade I–II	657 (66.8)	115 (71.4)	255 (66.8)	100 (70.9)	187 (62.3)	0.149
Vascular involvement (any extent), according to CT	148 (15.8)	24 (15.8)	42 (11.4)	23 (16.3)	59 (21.5)	0.007*
Neoadjuvant therapy in patients with PDAC	81 (13.3)	3 (3.3)	21 (9.1)	20 (21.3)	37 (19.5)	<0.001*
**ua-FRS**						0.074
Low	4 (0.5)	0 (0)	2 (0.6)	2 (1.6)	0 (0)	
Intermediate	239 (28.4)	46 (33.1)	106 (29.3)	40 (31.2)	47 (22.1)	
High	599 (71.1)	93 (66.9)	254 (70.2)	86 (67.2)	166 (77.9)	
Texture, soft	658 (71.7)	110 (76.9)	266 (72.7)	84 (65.1)	198 (70.7)	0.173
Pancreatic duct diameter (mm), median (i.q.r.)	3 (2–5)	4 (2–6)	3 (2–5)	3 (2–5)	3 (2–4)	0.012*
Operating time (min), median (i.q.r.)	367 (310–443)	420 (347–537)	360 (308–420)	349 (301–411)	369 (306–478)	<0.001*
Blood loss (ml), median (i.q.r.)	200 (100–450)	250 (100–5000	200 (100–438)	200 (100–400)	220 (100–500)	0.070
Conversion	101 (10.1)	35 (21.7)	39 (10.0)	4 (2.8)	23 (7.5)	<0.001*
Vascular resection performed	76 (7.6)	12 (7.5)	32 (8.2)	11 (7.7)	21 (6.8)	0.924

Values are *n* (%) unless otherwise indicated. *Statistically significant. RPD, robotic pancreatoduodenectomy; i.q.r., interquartile range; PDAC, pancreatic ductal adenocarcinoma; ua-FRS, updated alternative fistula risk score.

**Table 2:** Outcomes

Variable	Overall, RPD (*n* = 1000)	Phase 1, 1–15 RPD (*n* = 161)	Phase 2, 16–62 RPD (*n* = 389)	Phase 3, 63–84 RPD (*n* = 143)	Phase 4, >84 RPD (*n* = 307)	*P*
Major morbidity (CD complications grade ≥III)	408 (41.3)	64 (39.8)	151 (38.8)	65 (45.5)	128 (43.5)	0.428
In-hospital/30-day mortality	39 (3.9)	3 (1.9)	17 (4.4)	7 (4.9)	12 (3.9)	0.493
POPF grade B/C	244 (24.4)	30 (18.6)	95 (24.4)	34 (23.8)	85 (27.7)	0.193
POPF grade C	21 (2.1)	4 (2.5)	9 (2.3)	4 (2.8)	4 (1.3)	0.685
PPH grade B/C	122 (12.2)	18 (11.2)	52 (13.4)	15 (10.5)	37 (12.1)	0.790
PPH grade C	77 (7.7)	14 (8.7)	33 (8.5)	9 (6.3)	21 (6.8)	0.736
DGE grade B/C	224 (22.4)	52 (32.3)	88 (22.6)	22 (15.4)	62 (20.2)	0.003*
DGE grade C	101 (10.1)	18 (11.2)	45 (11.6)	11 7.7)	27 (8.8)	0.477
Bile leakage grade B/C	81 (8.1)	12 (7.5)	32 (8.2)	17 (11.9)	20 (6.5)	0.273
Bile leakage grade C	14 (1.4)	5 (3.1)	5 (1.3)	3 (2.1)	1 (0.3)	0.088
Reoperation	89 (8.9)	16 (9.9)	44 (11.3)	14 (9.8)	15 (4.9)	0.026*
Duration of hospital stay (days), median (i.q.r.)	11 (7–20)	12 (8–20)	11 (7–19)	10 (7–20)	10 (7–20)	0.035*
Readmission	213 (23)	40 (25.8)	81 (22.3)	23 (16.9)	69 (25.5)	0.206
R0 resection†	444 (69.1)	75 (72.8)	195 (72.2)	61 (66.3)	113 (63.5)	0.182
Histopathology						0.001*
PDAC	297 (29.7)	53 (32.9)	115 (29.5)	46 (32.6)	83 (27)	
Cholangiocarcinoma	143 (14.3)	21 (13)	57 (14.7)	21 (14.7)	44 (14.3)	
Ampullary cancer	165 (16.5)	27 (16.8)	78 (20.1)	11 (7.7)	49 (16)	
Duodenal cancer	48 (4.8)	6 (3.7)	23 (5.9)	12 (8.4)	7 (2.3)	
Neuroendocrine neoplasm	66 (6.6)	8 (5)	17 (4.4)	8 (5.6)	33 (10.7)	
IPMN, SPN, MCN	127 (12.7)	14 (8.7)	51 (13.1)	20 (14)	42 (13.7)	
Other/unknown	154 (15.4)	32 (19.9)	48 (12.4)	25 (17.5)	49 (15.9)	

Values are *n* (%) unless otherwise indicated. *Statistically significant. †For PDAC. RPD, robotic pancreatoduodenectomy; CD, Clavien–Dindo; POPF, postoperative pancreatic fistula; PPH, postoperative pancreatic haemorrhage; DGE, delayed gastric emptying; i.q.r., interquartile range; PDAC, pancreatic ductal adenocarcinoma; IPMN, intraductal papillary mucinous neoplasm; SPN, solid pseudopapillary neoplasm; MCN, mucinous cystic neoplasm.

### Nationwide improvements

**Fig. 3:**
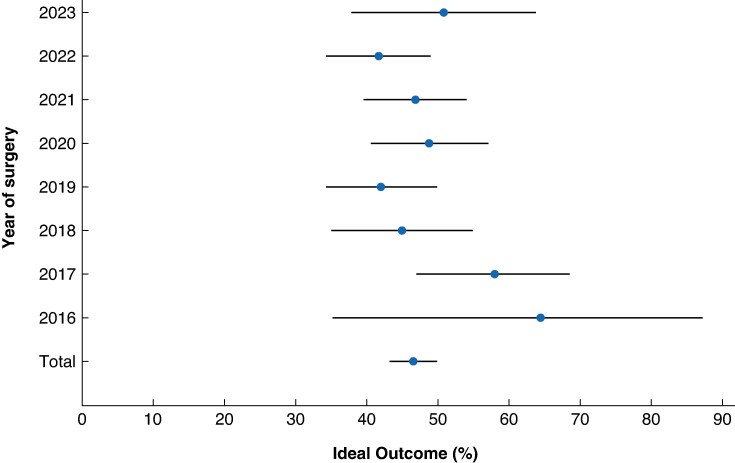
Funnel plot of nationwide annual rate of Ideal Outcome after RPD RPD, robotic pancreatoduodenectomy.

When comparing nationwide outcomes after RPD between the four phases, five outcomes demonstrated improvement: median operating time (420, 360, 349, and 369 min respectively; *P* < 0.001), conversion rate (21.7%, 10.0%, 2.8%, and 7.5% respectively; *P* < 0.001), rate of DGE of grade B/C (32.3%, 22.6%, 15.4%, and 20.2% respectively; *P* = 0.003), reoperation rate (9.9%, 11.3%, 9.8%, and 4.9% respectively; *P* = 0.026), and median duration of hospital stay (12 (i.q.r. 8–20), 11 (i.q.r. 7–19), 10 (i.q.r. 7–20), and 10 (i.q.r. 7–20) days respectively; *P* = 0.035). Between the four phases, no significant differences were observed for major morbidity and in-hospital/30-day mortality (*Table 2*), and no significant differences were observed for Ideal Outcome (45.9%, 46.9%, 46.8%, and 46.1%; *P* = 0.996). Also, when calculated per year, the nationwide rate of Ideal Outcome remained stable; *P* = 0.158 (*Fig. 3*).

### Outcomes between all centres

When combining all data per centre, no significant difference in overall in-hospital/30-day mortality was found between centres (*P* = 0.699).

### Outcomes in the most experienced centres

**Fig. 4:**
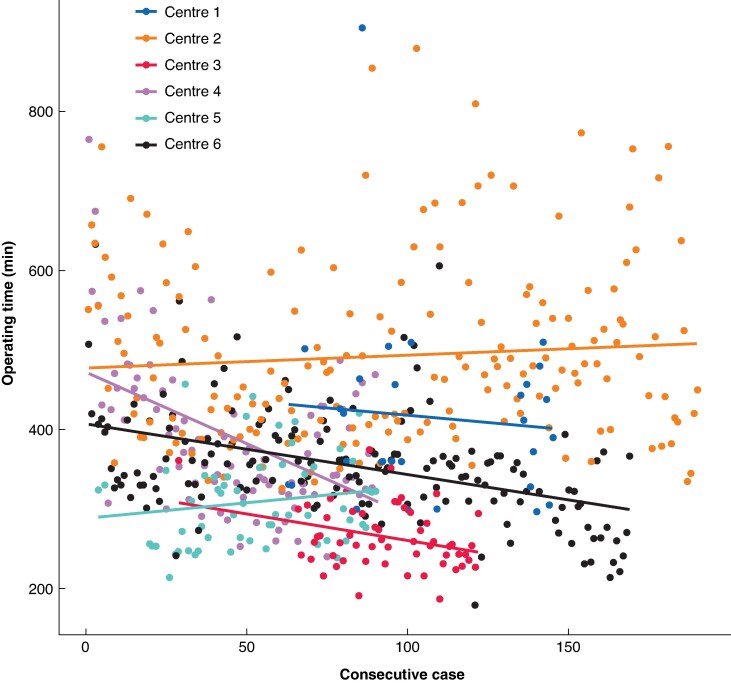
Scatter plot of trend in operating time of RPD in the six most experienced centres RPD, robotic pancreatoduodenectomy.

**Fig. 5:**
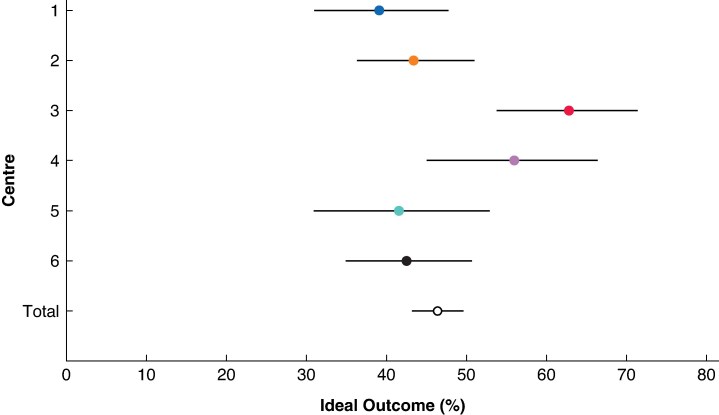
Funnel plot of rate of Ideal Outcome after RPD in the six most experienced centres RPD, robotic pancreatoduodenectomy.

Six centres fulfilled the definition of ‘most experienced centres’, as these included patients in all four learning curve phases. When assessing the five outcomes that had shown nationwide improvement per individual centre in the most experienced centres, three of the six centres showed significant improvement in operating time and one of the six centres showed significant improvement in reoperation rate. In these centres, the trends in operating time for RPD mostly showed a downward trend, but differed widely (*Fig. 4*). Moreover, the largest difference in operating time was seen between phase 1 and the other phases: phase 1, 481 (95% c.i. 446 to 517) min; phase 2, 377 (95% c.i. 364 to 390) min; phase 3, 359 (95% c.i. 342 to 374) min; and phase 4, 402 (95% c.i. 385 to 419) min (*P* < 0.001). The mean rate of Ideal Outcome was 47% (*Fig. 5*). Furthermore, in the most experienced centres (2 centres that performed ≥150 RPD), the conversion rate was <3%.

### Sensitivity analysis in low-risk (PDAC) patients

After excluding all patients with non-PDAC, significant differences during the four phases were observed for operating time (452, 384, 350, and 400 min respectively; *P* = 0.002), conversion rate (26.4%, 15.7%, 2.2%, and 7.2% respectively; *P* = 0.001), major morbidity (26.4%, 24.3%, 34.8%, and 42.3% respectively; *P* = 0.049), and rate of postoperative pancreatic fistula of grade B/C (1.9%, 7.0%, 8.7%, and 15.7% respectively; *P* = 0.036). Moreover, no significant differences were observed between the four phases for DGE grade B/C, postoperative pancreatic haemorrhage grade B/C, bile leakage grade B/C, and in-hospital/30-day mortality. See *Table S1*.

### Sensitivity analysis in low/medium-risk patients using the ua-FRS^16^

In patients with low/medium risk for POPF using the ua-FRS, significant differences during the four phases were observed for operating time (464, 387, 360, and 358 min respectively; *P* = 0.008), conversion rate (26.1%, 12.0%, 4.8%, and 4.3% respectively; *P* = 0.004), rate of bile leakage grade B/C (2.2%, 9.3%, 0.0%, and 2.1% respectively; *P* = 0.046), and median duration of hospital stay (15, 10, 8, and 8 days respectively; *P* < 0.001). See *Table S2* for more details. *Table S3* reports outcomes in high-risk patients using the ua-FRS.

## Discussion

This study found nationwide improvements for operating time, conversion rate, rate of DGE of grade B/C, reoperation rate, and duration of hospital stay for RPD. Notably, the rate of major morbidity and the rate of in-hospital/30-day mortality remained stable during the 7-year study interval. No differences in the rate of Ideal Outcome were found between centres.

Nationwide studies on RPD in the four phases of the learning curve are currently lacking. A National Cancer Database (NCD) analysis that included 799 patients after RPD (2010–2016) reported comparable results for postoperative mortality (3.1% NCD *versus* 3.9%) and the conversion rate (15.0% NCD *versus* 10.1%), but the readmission rate was higher in the present study (10.1% NCD *versus* 23%)^17^.

It is interesting to speculate whether, and to what extent, outcomes after RPD could further improve on a nationwide level in the coming years. When comparing the results of the present study with the largest Western single-centre experience of 500 RPD reported by the UPMC, there clearly seems room for further improvement regarding major morbidity (24% UPMC *versus* 41.3%), 30-day/in-hospital mortality (1.4% UPMC *versus* 3.9%), and postoperative pancreatic fistula grade B/C (7.8% UPMC *versus* 24.4%)^7^. It is important to note that this single-centre study reported outcomes from a single learning curve, whereas the present study includes learning curves from 13 centres^6,17^. Previous single-centre studies from the Netherlands also reported good results^18,19^, albeit not at a level of proficiency reported by the Pittsburgh group, which will require further experience, continuous assessment, and transatlantic collaboration.

When assessing the five outcomes with improvements among individual centres, some differences between centres became apparent. This is perhaps best illustrated by the operating time scatter plot (*Fig. 4*), demonstrating striking differences in operating time between the six most experienced centres. This is especially interesting, as all of these centres participated in the same training programme (that is the LAELAPS-3 training programme). These differences are most likely explained by differences in local practice, for instance regarding surgical training. In fact, the centre with the longest operating times trained five fellows as RPD console surgeons, whereas this was not done in the other centres. The scatter plot also demonstrates that a plateau has not yet been reached by any of the centres, suggesting that operating times may continue to improve. This is supported by the 2024 multicentre randomized trial from China that demonstrated shorter operating times for RPD compared with the open approach (245 *versus* 298 min; *P* = 0.001)^2^. Although operating time in itself does not have a clinically relevant influence on patient outcomes, it does give an insight into the ongoing development of RPD as a routine procedure.

It is important to realize that patients were heavily selected for RPD in this study. However, patient selection criteria were uniform, due to the LAELAPS-3 training programme, and did not change notably over the four phases. The conversion rate in phase four (>84 RPD) was 7.5% and thus relatively high. However, in the sensitivity analysis for the two centres that performed ≥150 RPD, the conversion rate was <3% (1.0% and 2.9%); this highlights that a plateau phase has not yet been reached in most centres. Furthermore, the negative correlation between the learning curve and the conversion rate persisted, suggesting that the 7.5% rate observed during the mastery phase may be a coincidental finding, from which no definitive conclusions can be drawn. The rate of patients with a high ua-FRS increased somewhat from 66.9% in phase 1 to 77.9% in phase 4. Furthermore, the rates of major morbidity, in-hospital/30-day mortality, postoperative pancreatic fistula of grade B/C, post-pancreatectomy haemorrhage of grade B/C, and Ideal Outcome remained stable. These findings also confirm the success of the training programme, facilitating the safe introduction of RPD in the Netherlands^7^.

In the present study, the rate of postoperative pancreatic fistula of grade B/C was 24.4%. This is lower than the 37.9% reported in a phase 2b RCT (the EUROPA trial) from Heidelberg^1^, but much higher than the 7.8% reported by the UPMC^7^. This may partly be explained by the multicentre design of the present study and the fact that approximately two-thirds of the patients had a high ua-FRS. Moreover, it could also have been influenced by the proactive detection and treatment of complications as implemented by the PORSCH trial during the same study interval^20^. This intervention might have increased the rate of detection of postoperative pancreatic fistula, due to increased use of percutaneous drainage, but may also have played a role in the decrease in postoperative pancreatic fistula of grade C to 0%.

Several limitations need to be considered when assessing the results of the present study. First, this study describes outcomes in a rather well-trained group of centres. Therefore, the findings may not be applicable to centres that start performing RPD outside a dedicated training programme. The LEARNBOT training programme is now available for high-volume European centres. Second, variations in general patient treatment practices over time might have impacted the learning curves and surgical outcomes. Third, although there were no differences in Ideal Outcome when assessing individual centres, a type II error cannot be excluded, as the sample sizes were small in some centres^20^. More data will have to be collected over an extended interval to assess whether improvements can also be identified within individual centres. Fourth, mortality data are only collected up to 30 days/in hospital, as this is the current standard in the Dutch auditing system. A start has been made to increase this to 90 days. Fifth, no data on common bile duct diameters, previous complicated upper abdominal surgery/disease, or whether the hepatic artery originates from the superior mesenteric artery have been collected, preventing application of the ISGPS complexity and experience grading system for minimally invasive pancreatoduodenectomy and the PD-ROBOSCORE for RPD in this study^21,22^. However, sub-analyses in patients with PDAC and in low/medium-risk patients using the ua-FRS were performed. The main strength is that this study is the largest nationwide multicentre study on outcomes of consecutive RPD within and beyond the initial learning curve, providing important insights into the effects of RPD during the extended implementation interval in a real-world, nationwide clinical situation. Also, the present study included all RPD procedures performed in all 13 centres, including the very first patients who are not infrequently excluded from reports.

## Collaborators

Freek Daams and Geert Kazemier (Amsterdam UMC, Amsterdam, The Netherlands); Khe Tran and Roel Haen (Erasmus MC, Rotterdam, The Netherlands); Alexander Vahrmeijer and Volkert Huurman (Leiden Universiteit Medisch Centrum, Leiden, The Netherlands); Robert Jan Schipper (Catharina Hospital, Eindhoven, The Netherlands); Ronald van Dam (Maastricht UMC, Maastricht, The Netherlands); Jennifer Schreinemakers (Amphia Hospital, Breda, The Netherlands); Lieke Brouwer-Hol and Brigitte C. M. Haberkorn (Maasstad Hospital, Rotterdam, The Netherlands); Mike Liem (Medisch Spectrum Twente, Enschede, The Netherlands); Wouter te Riele and Jeroen Hagendoorn (UMC Utrecht, Utrecht, The Netherlands); Martijn Stommel (Radboud UMC, Nijmegen, The Netherlands); Tom M. Karsten (OLVG, Amsterdam, The Netherlands).

## Supplementary Material

znaf210_Supplementary_Data

## Data Availability

Data are available from the corresponding author upon reasonable request.
