# Supplementary material for: Nationwide outcomes of 1000 robotic pancreatoduodenectomies across the four phases of the learning curve
Source: Br J Surg. 2025 Nov 6;112(11):znaf210. doi: 10.1093/bjs/znaf210 (PMC12596432; doi:10.1093/bjs/znaf210)
Supplement: znaf210_Supplementary_Data [file znaf210_supplementary_data.docx]

**Title: “Nationwide outcomes of 1000 robot pancreatoduodenectomies across the four phases of the learning curve”**

Authors: Anouk MLH Emmen, MD*1,2, Bram LJ van den Broek, MD*3, Tessa E Hendriks, MD1,2,4,5, Olivier R Busch, MD, PhD1,2, Bert A Bonsing, MD, PhD4, Marie L. Cappelle, MD, PhD3, Peter-Paul LO Coene, MD, PhD6, Sebastiaan Festen, MD, PhD7, Erwin van der Harst, MD, PhD6, Ignace HJT de Hingh, MD, PhD8, Cees JHM van Laarhoven, MD, PhD9, Daan J Lips, MD, PhD10, Joost Sprakel, MD10, Misha DP Luyer, MD, PhD8, J Sven D Mieog, MD, PhD4, Hjalmar C van Santvoort, MD, PhD11, George van der Schelling, MD, PhD12, J.H. Wijsman, MD, PhD 12, G.A. Patijn, MD, PhD13, Roeland F de Wilde, MD, PhD3, Maurice JW Zwart, MD, PhD1,2, W.J.M. Derksen, MD, PhD11, I Quintus Molenaar, MD, PhD#,11, Bas Groot Koerkamp, MD, PhD#,3, Marc G Besselink, MD, PhD#1,2, for the Dutch Institute for Clinical Auditing and the Dutch Pancreatic Cancer Group

1. Amsterdam UMC, location University of Amsterdam, Department of Surgery, the Netherlands

2. Cancer Center Amsterdam, Amsterdam, the Netherlands

3. Department of Surgery, Erasmus MC Cancer institute, Erasmus University Medical Centre, Rotterdam, the Netherlands

4. Department of Surgery, Leiden University Medical Centre, Leiden, the Netherlands

5. Dutch Institute for Clinical Auditing, Leiden, the Netherlands

6 Department of Surgery, Maasstad hospital, Rotterdam, the Netherlands

7. Department of Surgery, OLVG, Amsterdam, the Netherlands

8. Department of Surgery, Catharina Hospital, Eindhoven, the Netherlands

9. Department of Surgery, Radboud UMC, Nijmegen, the Netherlands

10. Department of Surgery, Medisch Spectrum Twente, Enschede, the Netherlands

11. Department of Surgery, Regional Academic Cancer Centre Utrecht, UMC Utrecht Cancer Center & St Antonius, the Netherlands

12. Department of Surgery, Amphia hospital, Breda, the Netherlands

13. Department of Surgery, Isala Zwolle, the Netherlands

**Corresponding author.**

Prof. Marc G Besselink, MD MSc PhD

Amsterdam UMC, location University of Amsterdam

Department of Surgery, Cancer Center Amsterdam

Tel: +31-20-4444400

Email: [m.g.besselink@amsterdamUMC.nl](mailto:m.g.besselink@amsterdamUMC.nl)

During the review process: Anouk Emmen, MD ([a.emmen@amsterdamUMC.nl](mailto:a.emmen@amsterdamUMC.nl))

X/Twitter: **@AnoukEmmen, @MarcBesselink, @Abuhilal9Abu**

**Supplementary Materials - Index**

| **Supplementary Figures and Tables** |  |
| --- | --- |
| Supplementary Table 1: Outcomes in patients with PDAC | *pag. 3* |
| Supplementary Table 2. Outcomes in patients with low/medium risk using the ua-FRS | *pag. 4* |
| Supplementary Table 3: Outcomes in patients with high-risk using the ua-FRS | *pag. 5* |

**Supplementary Tables**

| **Supplementary Table 1. Outcomes in patients with PDAC** | | | | | | |
| --- | --- | --- | --- | --- | --- | --- |
| **Variable** | **Overall**  **297 RPD** | **Phase 1**  **1-15 RPD**  **N=53** | **Phase 2**  **16-62 RPD**  **N=115** | **Phase 3**  **63-84 RPD**  **N=46** | **Phase 4**  **>84 RPD**  **N=83** | **P value** |
| Operative time, min, median [IQR] | 212 [386-472] | 452 [368-592] | 384 [325-444] | 350 [275-405] | 400 [290-491] | **0.002** |
| Blood loss, mL, median [IQR] | 300 [150-500] | 300 [200-1050] | 275 [150-600] | 250 [100-400] | 300 [150-513] | 0.446 |
| Conversion, n (%) | 39 (13.1) | 14 (26.4) | 18 (15.7) | 1 (2.2) | 6 (7.2) | **0.001** |
| Major morbidity (CD grade ≥3), n (%) | 91 (30.6) | 14 (26.4) | 28 (24.3) | 16 (34.8) | 33 (42.3) | **0.049** |
| In-hospital/30-day mortality, n (%) | 11 (3.7) | 1 (1.9) | 2 (1.7) | 3 (6.5) | 5 (6.0) | 0.260 |
| POPF grade B/C, n (%) | 26 (8) | 1 (1.9) | 8 (7.0) | 4 (8.7) | 13 (15.7) | **0.036** |
| PPH grade B/C, n (%) | 29 (9.8) | 3 (5.7) | 12 (10.4) | 5 (10.9) | 9 (10.8) | 0.742 |
| DGE grade B/C, n (%) | 50 (16.8) | 9 (17) | 17 (14.8) | 5 (10.9) | 19 (22.9) | 0.297 |
| Bile leakage grade B/C, n (%) | 16 (5.4) | 1 (1.9) | 8 (7.0) | 1 (2.2) | 6 (7.2) | 0.346 |
| Reoperation, n (%) | 18 (6.1) | 2 (3.8) | 7 (6.1) | 5 (10.9) | 4 (4.8) | 0.461 |
| Hospital stay, days, median [IQR] | 9 [6-16] | 12 [8-17] | 9 [6-14] | 8 [6-13] | 9 [6-18] | 0.074 |
| Readmission, n (%) | 49 (17.3) | 7 (13.7) | 20 (18.2) | 7 (15.9) | 15 (19.2) | 0.835 |
| *CD: Clavien Dindo, POPF: postoperative pancreatic fistula, PPH: postoperative pancreatic hemorrhage, DGE: delayed gastric emptying, IQR: inter quartile range.* | | | | | | |

| **Supplementary Table 2. Outcomes in patients with low/medium risk using the ua-FRS** | | | | | |
| --- | --- | --- | --- | --- | --- |
| **Variable** | **Phase 1**  **1-15 RPD**  **N=46** | **Phase 2**  **16-62 RPD**  **N=108** | **Phase 3**  **63-84 RPD**  **N=42** | **Phase 4**  **>84 RPD**  **N=47** | **P value** |
| Operative time, min, median [IQR] | 464 [395 - 555] | 387 [325 - 442] | 360 [318 - 429] | 358 [308 - 414] | **0.008** |
| Blood loss, mL, median [IQR] | 250 [200 - 700] | 200 [100 - 488] | 200 [100 - 400] | 200 [100 - 300] | 0.130 |
| Conversion, n(%) | 12 (26.1) | 13 (12.0) | 2 (4.8) | 2 (4.3) | **0.004** |
| Major morbidity (CD grade ≥3), n (%) | 17 (37.0) | 33 (30.6) | 15 (35.7) | 9 (19.1) | 0.230 |
| In-hospital/30-day mortality, n (%) | 1 (2.2) | 1 (0.9) | 1 (2.4) | 1 (2.1) | 0.895 |
| POPF grade B/C, n (%) | 4 (8.7) | 12 (11.1) | 3 (7.1) | 4 (8.5) | 0.876 |
| PPH grade B/C, n (%) | 4 (8.7) | 12 (11.1) | 2 (4.8) | 4 (8.5) | 0.678 |
| DGE grade B/C, n (%) | 15 (32.6) | 21 (19.4) | 6 (14.3) | 6 (12.8) | 0.071 |
| Bile leakage grade B/C, n (%) | 1 (2.2) | 10 (9.3) | 0 (0.0) | 1 (2.1) | **0.046** |
| Reoperation, n (%) | 5 (10.9) | 10 (9.3) | 3 (7.1) | 0 (0.0) | 0.164 |
| Hospital stay, days, median [IQR] | 15 [10 - 27] | 10 [7 - 18] | 8 [6 - 12] | 8 [6 - 10] | **<0.001** |
| Readmission, n (%) | 10 (22.2) | 23 (21.9) | 8 (19.0) | 7 (15.9) | 0.841 |
| R0 resection*, n (%) | 10 (47.6) | 28 (58.3) | 10 (45.5) | 8 (57.1) | 0.708 |
| *CD: Clavien Dindo, POPF: postoperative pancreatic fistula, PPH: postoperative pancreatic hemorrhage, DGE: delayed gastric emptying, IQR: inter quartile range, * in case of PDAC* | | | | | |

| **Supplementary Table 3. Outcomes in patients with high-risk using the ua-FRS** | | | | | |
| --- | --- | --- | --- | --- | --- |
| **Variable** | **Phase 1**  **1-15 RPD**  **N=93** | **Phase 2**  **16-62 RPD**  **N=254** | **Phase 3**  **63-84 RPD**  **N=86** | **Phase 4**  **>84 RPD**  **N=166** | **P value** |
| Operative time, min, median [IQR] | 420 [347 - 511] | 352 [296 - 411] | 342 [297 - 402] | 325 [277 - 394] | **<0.001** |
| Blood loss, mL, median [IQR] | 225 [100 - 425] | 200 [100 - 400] | 200 [100 - 390] | 220 [100 - 500] | 0.348 |
| Conversion, n(%) | 18 (19.4) | 24 (9.4) | 2 (2.3) | 14 (8.4) | **0.001** |
| Major morbidity (CD grade ≥3), n (%) | 39 (41.9) | 108 (42.5) | 45 (52.3) | 77 (46.4) | 0.395 |
| In-hospital/30-day mortality, n (%) | 1 (1.1) | 16 (6.3) | 6 (7.0) | 7 (4.2) | 0.184 |
| POPF grade B/C, n (%) | 24 (25.8) | 72 (28.3) | 28 (32.6) | 53 (31.9) | 0.653 |
| PPH grade B/C, n (%) | 13 (14.0) | 38 (15.0) | 13 (15.1) | 22 (13.3) | 0.962 |
| DGE grade B/C, n (%) | 28 (30.1) | 61 (24.0) | 16 (18.6) | 36 (21.7) | 0.291 |
| Bile leakage grade B/C, n (%) | 9 (9.7) | 22 (8.7) | 14 (16.3) | 14 (8.4) | 0.188 |
| Reoperation, n (%) | 10 (10.8) | 31 (12.2) | 10 (11.6) | 11 (6.6) | 0.309 |
| Hospital stay, days, median [IQR] | 11 [8 - 17] | 12 [7 - 21] | 12 [7 - 23] | 11 [7 - 25] | 0.970 |
| Readmission, n (%) | 25 (27.2) | 54 (23.1) | 13 (16.5) | 46 (30.3) | 0.110 |
| R0 resection*, n (%) | 21 (80.8) | 32 (57.1) | 13 (65.0) | 23 (71.9) | 0.171 |
| *CD: Clavien Dindo, POPF: postoperative pancreatic fistula, PPH: postoperative pancreatic hemorrhage, DGE: delayed gastric emptying, IQR: inter quartile range, * in case of PDAC* | | | | | |
